# Neural Substrates and Models of Omission Responses and Predictive Processes

**DOI:** 10.3389/fncir.2022.799581

**Published:** 2022-02-01

**Authors:** Alessandro Braga, Marc Schönwiesner

**Affiliations:** ^1^Institute of Biology, Faculty of Life Sciences, University of Leipzig, Leipzig, Germany; ^2^International Max Plank Research School, Max Plank Institute for Human Cognitive and Brain Sciences, Leipzig, Germany; ^3^International Laboratory for Research on Brain, Music, and Sound (BRAMS), Université de Montréal, Montreal, QC, Canada

**Keywords:** corollary discharge, mismatch negativity, predictive coding, feedforward inhibition, stimulus specific adaptation (SSA), interactionism, animal research

## Abstract

Predictive coding theories argue that deviance detection phenomena, such as mismatch responses and omission responses, are generated by predictive processes with possibly overlapping neural substrates. Molecular imaging and electrophysiology studies of mismatch responses and corollary discharge in the rodent model allowed the development of mechanistic and computational models of these phenomena. These models enable translation between human and non-human animal research and help to uncover fundamental features of change-processing microcircuitry in the neocortex. This microcircuitry is characterized by stimulus-specific adaptation and feedforward inhibition of stimulus-selective populations of pyramidal neurons and interneurons, with specific contributions from different interneuron types. The overlap of the substrates of different types of responses to deviant stimuli remains to be understood. Omission responses, which are observed both in corollary discharge and mismatch response protocols in humans, are underutilized in animal research and may be pivotal in uncovering the substrates of predictive processes. Omission studies comprise a range of methods centered on the withholding of an expected stimulus. This review aims to provide an overview of omission protocols and showcase their potential to integrate and complement the different models and procedures employed to study prediction and deviance detection.This approach may reveal the biological foundations of core concepts of predictive coding, and allow an empirical test of the framework’s promise to unify theoretical models of attention and perception.

## Introduction

The ability to learn associations between stimuli and behavior is the main selective pressure on central nervous systems across species and appears to be among the core functions of the neocortex (Badcock et al., 2019). How do cortical processes track which associations have been acquired, and which need to be acquired? The predictive processing framework argues that if the brain can predict the incoming sensory input, that is, the sensory consequences of behavior, then no learning needs to take place, because it already happened (Friston, 2003; Keller and Mrsic-Flogel, 2018; Kirchhoff and Robertson, 2018). Vice versa, if a prediction is violated then the internal predictive model needs to be updated. By constant comparison between internal models and sensory input, learning minimizes discrepancies between expectations and outcomes. This framework encompasses several theories that assume prediction as a core cortical function, actively performed by change-detecting circuits. These theories have an appealing explanatory power, and suggest organic accounts of diverse phenomena and correlates of human cognition (Hutchinson and Barrett, 2019), including modulation of stimulus perception (Cardoso-Leite et al., 2010), intentional binding (Haggard et al., 2002), N1 attenuation, Mismatch Negativity (MMN), omission responses (Bendixen et al., 2012), gamma and beta band modulation (van Pelt et al., 2016), and schizophrenic and autistic symptomatology (Ford et al., 2014; Smith et al., 2021). Furthermore, the framework can explain fundamental functions, like attention and decision making, as being subserved by unitary and complementary mechanisms that act on the same substrates (Schröger et al., 2015; Burr, 2017). Because of its width and scope, the predictive processing framework can include multiple models of predictive processes like corollary discharge and deviance detection. This review provides a perspective on the compatibility of such models, at the substrate and mechanistic level provided by animal research, with a focus on the contribution of omission studies to our understanding of predictive mechanisms.

## Corollary Discharge: A Window on Predictive Substrates

**Figure 1:**
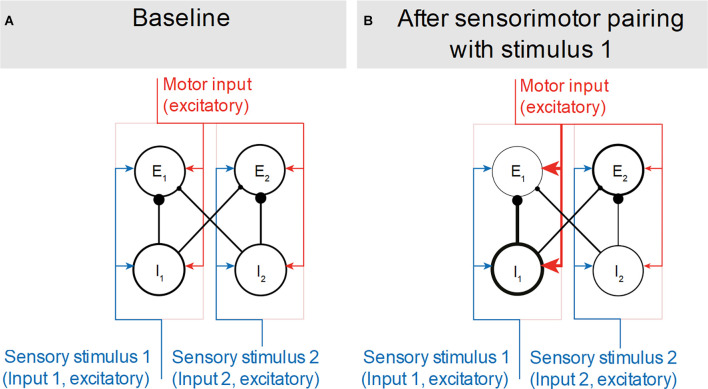
Model of corollary discharge microcircuitry before and after action-stimulus association. **(A)** In the networks’ baseline state, motor input (red arrows) results in inhibition of sensory responses in all excitatory populations (E_1,2_). Such generalized inhibition, independent from sensory input (blue arrows) is mediated by all inhibitory populations (I_1,2_). **(B)** After repeated paired presentations of motor and sensory input (i.e., stimulus 1), the motor input results in stimulus-specific inhibition for the paired sensory input. Specific inhibition is likely mediated by long-term potentiation of motor-to-sensory synapses (thick red arrows), which results in increased activation of I_1_ interneurons that are tuned to the action-paired stimulus. Circles indicate neural populations and lines indicate their connectivity. Arrows and filled circles indicate excitatory and inhibitory synapses, respectively. Thickness is used to indicate the strength of synaptic connectivity (arrows and filled circles) and stimulus response (circles and lines). Excitatory and inhibitory populations that respond preferentially to the same stimulus are grouped and surrounded by light pink rectangles.

Information on the physiology underlying predictive functions is necessary to test hypotheses about their mechanisms. Theories in the predictive processing framework are only fully testable when their substrate implementation is addressed (Kogo and Trengove, 2015; Heilbron and Chait, 2018). One example is the investigation of corollary discharge, a predictive mechanism by which motor processing exerts top-down modulation on sensory areas (Straka et al., 2018). Electrophysiology, calcium imaging, and optogenetics in rodents have demonstrated that, after learning a sensory-motor association, pyramidal projections from motor areas suppress the processing of action-associated sensory input (Rummell et al., 2016; Schneider et al., 2018) when the associated action is performed. This motor-associated sensory suppression is dependent on local interneuronal circuitry in sensory cortices (Figure 1) and it is observed across species, including humans, mice, and non-human primates (Schafer and Marcus, 1973; Eliades and Wang, 2003; Bäss et al., 2008; Schneider et al., 2018; Klaffehn et al., 2019; for a review of human studies, see Bendixen et al., 2012; Horváth, 2015; for a review focused on cortical substrates, see Reznik and Mukamel, 2019).

According to Schneider et al. (2018), the corollary discharge substrate includes neural populations that respond to specific stimuli and comprise reciprocally interconnected excitatory and inhibitory units. In its baseline state (Figure 1A) these interconnected stimulus-specific networks receive sensory and motor input to all units. The motor input causes generalized interneuron-mediated suppression of excitatory units during movement. If an action and a stimulus are paired repeatedly (Figure 1B), stimulus- and action-specific motor-to-sensory synapses are strengthened. After such learning, when the action is performed, generalized suppression is reduced and the pyramidal population responding to the paired stimulus is suppressed.

It has not been determined yet whether different interneuronal subpopulations have different roles in generalized vs. stimulus-specific inhibition of auditory pyramidal neurons during movement. Somatostatin (SST) interneurons are known to modulate sensory activity based on top-down intracortical input (for a review see Yavorska and Wehr, 2016). SST interneurons have narrower receptive fields than Parvalbumin (PV) interneurons (Li et al., 2015), which they inhibit (Ma et al., 2012; Cottam et al., 2013; Natan et al., 2017). SST-mediated feedforward inhibition of PV interneurons should be investigated during the association of an action and its sensory consequences. Such a study would allow assessment of whether the respective contribution of PV and SST populations to motor-driven sensory suppression changes as the action-stimulus association is acquired. Interneuron-type-dependent feedforward inhibition is also involved in deviance detection processes (Ross and Hamm, 2020), as detailed in the following section.

Independently from the different contributions of different interneuron populations, feedforward inhibition of auditory pyramidal neurons is a potential substrate for the “prediction” component of classic predictive processing models. Unexpected non-inhibited pyramidal activity constitutes a prediction error signal that drives synaptic plasticity between interneurons and their modulatory inputs (Schneider et al., 2018). This plasticity modifies future inhibition of pyramidal neurons, i.e., future predictions and prediction errors. If that is the case, then manipulation or disruption of synaptic plasticity and pyramidal-to-interneuron connectivity should affect responses to deviations from motor-sensory associations, such as the omission responses shown by SanMiguel et al. (2013b), besides affecting sensory suppression as already demonstrated by Schneider et al. (2018). This insight could apply also to canonical predictive processing models: prediction error is not exclusively a bottom-up signal transmitted by specific units (Kogo and Trengove, 2015; Heilbron and Chait, 2018; Walsh et al., 2020), rather, error signals and part of the prediction updating process can happen locally on the same synapses involved in the modulation of the stimulus response.

However, corollary discharge is limited to motor-to-sensory modulation and its mechanism is difficult to generalize to the wider scope of predictive processing. An open question is whether motor modulation of sensory activity constitutes a predictive process in itself, or whether it is integrated with ongoing, local processing of sensory regularity and deviation. For these purposes it is necessary to investigate the microcircuitry underlying other biomarkers of predictive processes, particularly in animal models, to assess the overlap of their substrates with the substrates of corollary discharge.

## Mmn as A Major Biomarker of Prediction

Most studies on predictive processing in humans have focused on the MMN, a brain potential elicited by any deviant that interrupts a stream of regular stimuli (Näätänen et al., 1982). This frontocentral, negative event-related potential component reflects pre-attentive detection of change in sensory streams (Schröger and Wolff, 1996). In the human auditory system, the MMN is observed in response to pitch, intensity, duration (Näätänen et al., 1993; Giard et al., 1995), location (Schröger and Wolff, 1996), and sequence deviants (Hofmann-Shen et al., 2020), among others. The MMN is reduced in several neurological disorders (Näätänen et al., 2014). For example, MMN reduction is a biomarker of schizophrenia, a disorder that involves dysfunctional predictive processing (Wacongne, 2016; Smith et al., 2021).

**Figure 2:**
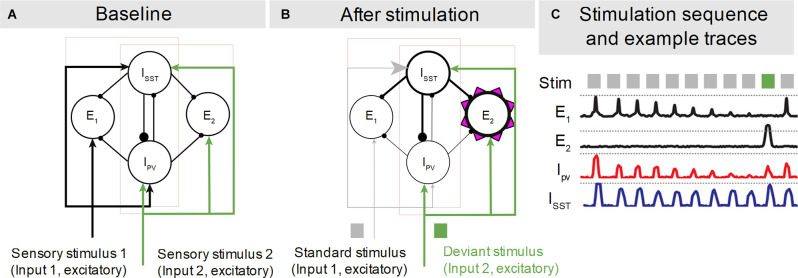
Hypothetical model of mismatch negativity microcircuitry based on electrophysiology and tracing studies in mice. **(A)** In its baseline state interconnected excitatory populations (E_1,2_) respond similarly to the stimuli (black and green bottom-up arrows) they are selective for, with no relevant modulation from local interneurons (which are not stimulus selective). **(B)** After repeated stimulation the standard-selective pyramidal population (E_1_) reduces its excitability due to inherited adaptation from the upstream sensory input, and the deviant-selective population (E_2_) increases its excitability due to NMDA-mediated (purple triangles) synaptic potentiation. Circles indicate neural populations and lines their connectivity. Arrows and filled circles indicate excitatory and inhibitory synapses, respectively. Thickness is used to indicate the strength of synaptic connectivity (arrows and filled circles) and stimulus response (circles and lines). I_SST_ and I_PV_ indicate somatostatin and parvalbumin interneurons, respectively. Excitatory and inhibitory populations that respond preferentially to the same stimuli are grouped and surrounded by light pink rectangles. Only the relevant connectivity is shown. **(C)** Typical activity traces for these neural populations as they transition from baseline to adapted activity (E_1_, I_PV_), including responses (E_2_) to a deviant during an oddball paradigm. Grey and green squares indicate the stimulus sequence. Adapted from Ross and Hamm (2020).

The mechanism of the MMN, as currently understood, involves at least two processes: a passive adaptive process (McEvoy et al., 1993; Jääskeläinen et al., 2004) that reduces the responsiveness of neural assemblies to repeated stimuli, called stimulus-specific adaptation (SSA, Ulanovsky et al., 2003; for a review see Nelken, 2014), and an active predictive process, sometimes called “genuine deviance detection” in this context (Näätänen et al., 1982, 2005; Schröger and Wolff, 1996). These distinct processes were revealed by the inclusion of control conditions for adaptation in oddball paradigms (Ruhnau et al., 2012). Such control paradigms have been translated to animal studies (Harms et al., 2016; Parras et al., 2017) and have not only shown that mismatch responses depend on both processes but also that adaptation and genuine deviance detection are not reciprocally exclusive and possibly share substrates. In particular, individual pyramidal neurons and interneurons respond differently to deviants and standards in oddball paradigms (Chen et al., 2015; Natan et al., 2015). Such responses are consistent with both deviance detection and SSA. Optogenetic manipulation showed that silencing PV interneurons enhances pyramidal responses to deviants and standards while silencing SST interneurons enhances responses to standards only (Natan et al., 2015). Another study, with simultaneous chemogenetic manipulation and electrophysiological measurement of visual cortex activity, demonstrated that SST interneurons are necessary for deviance detection, but their suppression does not affect the SSA component of mismatch responses in the local field potential (Hamm and Yuste, 2016). Intracranial studies in humans confirmed mixed contributions of deviance detection and adaptation to mismatch responses in auditory cortices (Ishishita et al., 2019). Furthermore, such studies provided indications that cortical processing is not spatially homogeneous (Hughes et al., 2001; Flinker et al., 2011; Fonken et al., 2019) and that adaptation and deviance detection responses can be differently distributed (Blenkmann et al., 2019; Ishishita et al., 2019), indicating the existence of specialized, interconnected sub-regions within the auditory stream for the processing of contextual information. Thus, animal studies make it possible to dissect the distinctive contributions of different interneuron populations to network-level SSA (Natan et al., 2017) and deviance detection mechanisms (Ross and Hamm, 2020) that are likely to underlie similar processes observed in humans. The precise role of each cell type is not yet understood. Mounting evidence indicates that both effects are at least partly produced by reciprocal interactions between interneurons, which affect pyramidal activity. Ross and Hamm (2020) proposed a mechanistic model of this circuitry (Figure 2).

According to Ross and Hamm (2020), the MMN substrate includes pyramidal populations that respond to specific stimuli and make reciprocal connections with PV and SST interneurons. Unlike Schneider et al. (2018), they assume that only pyramidal neurons receive exclusively stimulus-specific input and display stimulus-specific responses. Interneurons in their model are not necessarily stimulus selective. Interconnected excitatory and inhibitory units thus form stimulus-responsive, overlapping networks that exist in a baseline state (Figure 2A) that changes in response to repetitive stimulation (Figure 2C). These networks transition to a state (Figure 2B) in which the populations responding to the standard stimulus are adapted, and the populations responding to the deviant are primed for enhanced responses.

Repetitive stimulus presentation leads to SSA in the pyramidal population responsive to the standard stimulus, due to synaptic depression inherited from upstream synapses. Hence all populations responding to the standard stimulus decrease their response amplitude with repeated stimulation. However, the overall adaptation is less intense in SST interneurons, which stay more active throughout stimulus presentation, possibly due to concomitant short-term facilitation on SST interneuron synapses (Reyes et al., 1998) that could counterbalance inherited SSA. The input related to the deviant stimulus remains unadapted. The deviant-selective population displays enhanced responses, above the baseline level, due to SST interneurons silencing PV interneurons and consequently disinhibiting the non-adapted excitatory populations that respond to other stimuli than the standard. This disinhibition, in the deviant responding population, elicits the opening of NMDA receptors thus facilitating pyramidal activation in response to deviant input. This does not happen in standard-responsive excitatory neurons, due to their adapted state.

A distinctive feature of this model, which is based on experimental evidence from human and animal research, is that the generators of SSA and deviance detection overlap, and form complex excitatory/inhibitory networks (Askew and Metherate, 2016; Hennequin et al., 2017; Ross and Hamm, 2020). The results of animal mismatch response investigations are also consistent with notions of interneuron-mediated corollary discharge, mentioned earlier (Schneider et al., 2018). Several studies have found contextual and intermodal modulation of pyramidal activity to be dependent on interneuron-mediated inhibitory-disinhibitory control (Lee et al., 2013; Pi et al., 2013; Fu et al., 2014; Kuchibhotla et al., 2017; Natan et al., 2017; Phillips et al., 2017). Thus, from a predictive processing perspective, representation of regularity (prediction) is supported by adaptive processes while deviance detection (prediction error) relies on a combination of baseline state and synaptic facilitation. Thus, prediction and prediction error complement each other and are processed by the same circuitry. As for corollary discharge, this perspective is in contrast with the traditional formulation of prediction and prediction error as symmetrical flows of information.

Taken together, these results underline the importance of reciprocally wired interneurons as elements of circuits capable of complex computations, like those supporting corollary discharge and MMN. A currently open question is the extent of overlap in the circuitry that supports these deviance-related functions. This question could be addressed with protocols in which stimulus predictability is established both *via* sensory-motor association and *via* sensory stimulus regularity. The effects of each type of predictability on stimulus responses could be measured or modulated with circuital manipulation as implemented by Schneider et al. (2018), and their reliance on shared circuitry could thus be evaluated.

## Interactionist Perspectives for The Study of Predictive Processing

One possibility is that these integrative computations are supported by the same cortical circuitry, which could be generalized as predictive. The principle of a deviance detection circuit motif, which is replicated across the neocortex, is consistent with the notion of conservation of cortical microstructure across species and modalities (Douglas and Martin, 2004; Wang et al., 2010), and with the notion of a fundamental involvement of interneurons in the processing of stimuli according to context (Kuchibhotla et al., 2017; Lee et al., 2017; David, 2018). Such a circuit motif could accommodate a range of empirically informed models of local deviance detection (Schneider et al., 2018; Chien et al., 2019; Ross and Hamm, 2020) that rely on feedback and feedforward modulation of pyramidal output, mediated by interneuron populations with different dynamics.

Biologically realistic models are necessary to translate between human and non-human animal data, because they can, through simulations, answer the question of whether circuits discovered in non-human models can generate the responses observed in humans. In practice, mechanistic computational models of neuronal circuitry are made realistic by incorporating features of neural dynamics and connectivity that are derived mostly from animal research (Kohl et al., 2021). Model-fitting then allows the assessment of whether the modeled circuitry can produce observed human responses with realistic parameters, which provide further insight into the underlying processes. This type of model-driven interaction between animal and human research is needed to connect network structure, deviance detection phenomena, and their mechanistic explanations (Badre et al., 2015). However, the successful application of this approach to deviance detection and predictive processing requires a substantial amount of empirical data, which is still missing.

In particular, a significant differentiation of the roles of different neuronal populations in the generation of mismatch responses has been obtained only in the visual cortex (Hamm and Yuste, 2016). The role of vasopressin interneurons, which preferentially target other interneurons and mediate feedforward excitation, has not been studied in the context of deviance detection. A role of NMDA receptors and short-term plasticity in such cortical circuitry is assumed by most models, and they have proven crucial for deviance detection (Lee et al., 2018) and corollary discharge (Kort et al., 2017), but their precise function and extent of involvement in SSA (Farley et al., 2010; Chen et al., 2015) has yet to be determined.

Furthermore, sensory input has never been silenced in studies that targeted sensory cortices with optical imaging methods. This approach does not allow bottom-up stimulus representation, top-down modulation of local cortical activity, and response modulation performed by local excitatory-inhibitory circuitry to be fully distinguished. The advancements described in the previous section have been largely produced by techniques that can silence selected elements of circuitry. The equivalent of silencing techniques in behavioral studies of deviance detection is to silence the deviant stimulus. The neuronal responses to omitted, predictable stimuli are a special case of deviant responses called “omission responses” (Bendixen et al., 2012).

## Omission Responses: The Missing Protocol

**Figure 3:**
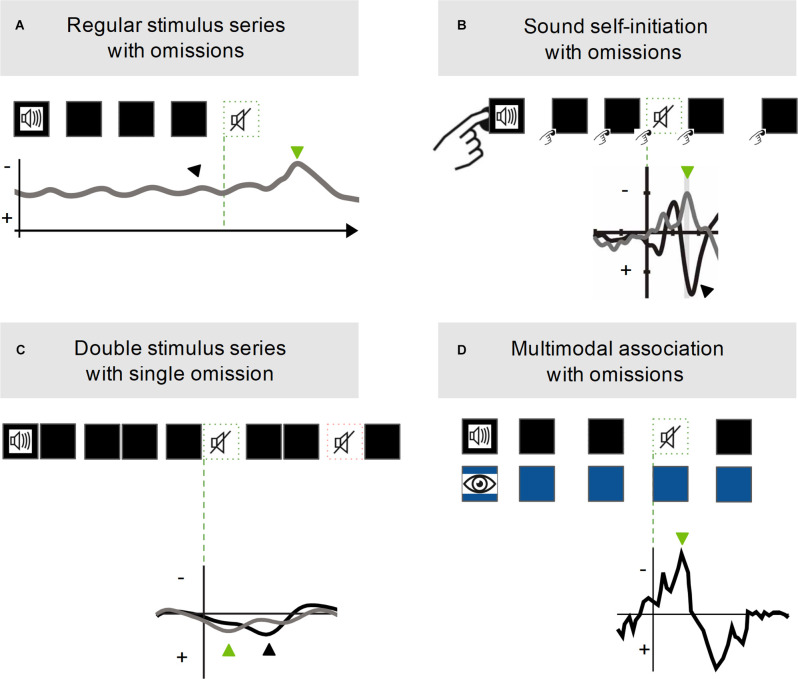
Summary of omission response protocols and response examples. **(A)** The omission protocol consists of a series of auditory stimuli (black squares) presented at regular intervals. The omission response (green arrow) is observed at the end of the stimulus train, at a fixed latency from the onset of the expected but missing stimulus (green dashed square). Adapted from Andreou et al. (2015). **(B)** The button-pressing symbol indicates that the auditory stimuli are self-initiated. This protocol consists of self-paced button pressing to which a stimulus is associated but randomly withheld. The omission response is observed when the action is performed but the action-associated stimulus is withheld. The black arrow indicates the response to the standard self-initiated stimulus. Adapted from SanMiguel et al. (2013a). **(C)** The orange dashed square indicates the omission of the first stimulus in a pair. This protocol consists of a series of stimulus pairs of which the first or second stimulus is randomly omitted. The omission response is observed when the second stimulus of the pair is omitted. Adapted from Bendixen et al. (2009). **(D)** The blue squares represent visual stimuli. This protocol consists of a series of paired presentations of a visual stimulus and an auditory stimulus, in which the auditory stimulus is randomly omitted. The omission response is observed in auditory areas when the auditory stimulus is omitted. Adapted from Stekelenburg and Vroomen (2015).

Omission responses can be understood as special cases of mismatch responses to an extreme stimulus deviation along the intensity dimension, that is, when an expected stimulus is not delivered. Therefore, omission responses offer a vantage point to study the activity of cortical circuitry at least partly decoupled from bottom-up input. Such a perspective allows better access to any endogenous neural activity that could be correlated with the generation of predictions (Schröger et al., 2015). In every protocol that establishes stimulus predictability, an omission response can be evoked by withholding the predicted stimulus. For instance, responses to the absence of stimuli can be observed in oddball protocols with silent oddballs (Karamürsel and Bullock, 1999; Busse and Woldorff, 2003), at the end of regular/rhythmic stimulus trains (Figure 3A; Andreou et al., 2015), with stimulus self-initiation protocols when an action-associated sensory stimulus is withheld (Figure 3B; SanMiguel et al., 2013b), and when other sensory-sensory associations, unimodal or multimodal, are established and one of the stimuli is withheld (Figures 3C,D; McIntosh et al., 1998; Hughes et al., 2001; Bendixen et al., 2009; den Ouden et al., 2009; Stekelenburg and Vroomen, 2015; van Laarhoven et al., 2017, 2020).

Omission responses observed with electrophysiology are characterized by a variety of components at different latencies, including early negative components (N1-like), mid latency negativities (MMN-like and N2-like), and later positive components (P3-like). These omission responses have been interpreted as representing a range of processes, which are not mutually exclusive. Bullock and colleagues studied omission responses extensively across species (Prechtl and Bullock, 1994; Ramón et al., 2001), modalities (Bullock et al., 1994; Karamürsel and Bullock, 1999), and methods (Bullock et al., 1993; Karamürsel and Bullock, 1994), and suggested that omission responses may be explained as a combination of temporal expectations and OFF responses to the termination of stimulus trains (Ramón et al., 2001). In particular, they distinguished between omission responses generated by fast rhythms, which are independent of attention and omission responses generated by slow rhythms, which are attention dependent (Karamürsel and Bullock, 1999; Ramón et al., 2001). “Fast” omission responses were characterized by fast and early components at a fixed latency from the time the missing stimulus was due. They hypothesized that such responses arise early in the human sensory pathway, and found them to be similar to those observed in retinas and optic nerves of animal models. Conversely, “slow” omission responses were characterized by a slow rising component and appeared to have been exclusively cortical. Hence, Karamürsel and Bullock (1999) hypothesized that the “slow” responses were related to higher-order cognitive processes. They further hypothesized that general excitatory/inhibitory homeostasis and rebound from inhibition could generate omission responses at different levels of the sensory pathway. According to Karamürsel and Bullock (1999), electrophysiological omission responses would thus reflect multiple similar mechanisms, with scalp-measured responses in humans being mostly cortical.

While the studies by Bullock and colleagues are based exclusively on electrophysiology and predate the predictive processing framework (Bullock et al., 1993, 1994; Karamürsel and Bullock, 1994, 1999; Prechtl and Bullock, 1994; Ramón et al., 2001), their conclusions are consistent with more recent conceptualizations of omission responses and could be considered as the first steps integrating the main conflicting accounts of omission responses: omission responses as a rebound from adaptation (May and Tiitinen, 2004, 2010; Thivierge and Cisek, 2011) and omission responses as prediction or prediction errors (den Ouden et al., 2009; Bendixen et al., 2012; Schröger et al., 2015; Heilbron and Chait, 2018). May and Tiitinen (2004) proposed that omission responses are the result of a rebound from adaptation in stimulus-responsive populations, entrained by the rhythm of past stimuli.

This perspective, however, does not account for omission responses to non-rhythmic protocols, for example, self-generation protocols (SanMiguel et al., 2013b). The experimental work of Bullock and colleagues offers a starting point for conciliation of such evidence with that for the involvement of entrainment (Andreou et al., 2015) in omission responses in rhythmic protocols (Bullock et al., 1994; Karamürsel and Bullock, 1999). This is particularly true of their observation that omission responses to different rhythms have different properties and could depend on different mechanisms, including rebound from adaptation. From this observation, they derive a dichotomy between high-level prediction responses, reliant on cognitive processes, and low-level prediction responses reliant on entrainment (Karamürsel and Bullock, 1999). Tiitinen’s account of omission responses considers the latter explanation sufficient for most commonly observed omission phenomena. An approach that considers both low-level and higher-level processes in the generation of omission responses, however, does not need to make them exclusive and unrelated. In fact, the dichotomy suggested by Bullock is equivalent to that of adaptation vs. genuine deviance detection accounts of MMN, in that it inspired research that went beyond the dichotomy. From that perspective, low-level processes, like SSA, are supported by the same networks as more complex forms of deviance detection, and SSA might even be necessary for deviance detection as suggested by Ross and Hamm (2020).

Omission responses and MMN are both responses to deviants, with prominent generators in the sensory cortex that depend on stimulus modality (Bendixen et al., 2012). Thus, considering also the relative uniformity of cortical architecture, it is plausible that omission responses and MMN rely on a similar combination of SSA and genuine deviance detection and possibly on the same local circuitry (Chien et al., 2019). Karamürsel and Bullock (1999) hypothesized “slow” and “fast” omission responses to be generated by possibly similar, but ultimately separate and non-overlapping, mechanisms, especially because of the differential modulatory effect of attention on the responses obtained with either stimulation protocol. However, considering the large neural population necessary to generate EEG-detectable responses, and the role of attention in enhancing brain responses to stimuli, the same MMN-generating circuitry could underlie both the “slow” and “fast” omission responses distinguished by Karamürsel and Bullock (1999). Accordingly, the “slow/fast” distinction could be a measurement artifact, with EEG being able to detect only responses to omissions in fast stimulus series, or responses to omissions enhanced by attentional or behavioral modulation.

Across human studies, omission responses obtained by employing slow-rhythm and non-rhythmic protocols require either an attention-driven increase in neuronal gain (Motz et al., 2013; Hernández and Hernández-Sánchez, 2017), multimodal projections to local circuitry (McIntosh et al., 1998; Nittono, 2005; den Ouden et al., 2009; SanMiguel et al., 2013a, b; Stekelenburg and Vroomen, 2015; van Laarhoven et al., 2017, 2020; Dercksen et al., 2020), or other forms of naturalistic (Lehmann et al., 2016) or behavioral relevance (Woerd et al., 2017; Aitken et al., 2020) of the omitted stimulus, in order to be detectable. In fact, attentional and top-down modulation of local circuitry can produce the synchronized activity of large neuronal populations necessary to produce detectable EEG responses to stimuli. In a few animal studies, the use of imaging methods that do not require large-scale activity, like calcium imaging, revealed omission-like responses to slow rhythms independent from attention or any other contextual factor (Sumbre et al., 2008; Li et al., 2017; Wang et al., 2018). This hypothesis is supported by at least one intracranial study in humans. Hughes et al. (2001) found omission responses to slow rhythms in the auditory cortex, independent from the attentional state, likely due to the higher resolution provided by intracranial measurements. Conversely, omission responses to fast rhythms are observable even without high resolution measurements or increased gain due to stimulus relevance. This difference might be due to entrainment and the higher overall stimulus energy injected into the circuit by fast stimulus presentation protocols (Zhang et al., 2013; Teschner et al., 2016). If that is the case, optical imaging in rodents should detect omission responses across a range of stimulation protocols (slow and fast), while EEG should detect omission responses only to fast rhythms unless attention, multimodal associations, or behaviorally relevant stimuli are involved.

Entrainment is one of the multiple ways to represent regularity (Sumbre et al., 2008; Fujioka et al., 2015; Obleser and Kayser, 2019) as part of low-level time tracking processes in the brain (Paton and Buonomano, 2018). These entrained representations of rhythm are prominent in early stations of the sensory pathway (Joris et al., 1994), and possibly when fast and regular stimuli are involved. The fact that EEG signals depend on the synchronized activity of large neuronal populations might explain why omission responses in fast-rhythm protocols can be observed in the absence of attention, unlike those in slow-rhythm protocols (Demarchi et al., 2019; Kirino et al., 2019; Okayasu et al., 2019). While the role of entrainment is speculative and beyond the scope of this review, the role of behavioral relevance in omission responses to non-rhythmic stimuli is supported by a large literature that is considered in more detail in the next section.

The variability of omission responses might also reflect a processing hierarchy. This concept echoes the seminal speculations of Karamürsel and Bullock (1999) on “different” omission responses representing higher or lower-level processes. A similar possibility is explored by Wacongne et al. (2011) with a protocol establishing stimulus predictability on the basis of both short-term stimulus history (local regularity) and block-wise, long-term stimulus history (global regularity). Early and late components of the responses to unexpected omissions were modulated differently depending on the interaction of the local and global rules. This result suggests that the complexity of omission responses could reflect different processing stages of deviation, from lower levels (violation of local regularity established by recent stimulus history) to higher levels (violation of global regularity established by stimulus patterns over blocks of stimuli).

## Omission Responses in Non-Rhythmic Protocols

Omission responses can be induced with protocols that do not rely on stimulus rhythmicity (Figures 3B–D), indicating that the underlying processes cannot be reduced to rebound effects caused by the interruption of rhythmic stimulation. Such protocols range from the fully non-rhythmic, relying on multimodal associations to establish stimulus predictability, to protocols that introduce jitter in the temporal predictability of the stimulus; however, jittered protocols do not necessarily elicit omission responses. The striking difference between studies that show omission responses to jittered, non-rhythmic stimulation, and studies that do not, is that the former establish some sort of behavioral relevance of the stimulus.

For instance, one recent study (Lehmann et al., 2016) elicited omission responses with a non-rhythmic protocol employing recorded syllables as stimuli. Furthermore, other studies that showed responses to the absence of a stimulus (Bullock et al., 1994; Jongsma et al., 2004; Todorovic et al., 2011; Chouiter et al., 2015), required the subjects to attend, detect, report, or predict the omissions. In contrast, passive listening studies in which a temporally irregular stimulus sequence is not attended, do not elicit responses to the omission of a stimulus (Takasaka, 1985; Andreou et al., 2015; Wang et al., 2018). Thus, it appears that behavioral relevance of the stimulus, whether naturalistic or induced by protocol, is necessary to elicit detectable and strong omission responses in irregular stimulus presentation protocols. These omission responses tend to resemble stimulus-evoked responses in shape and to surpass them in amplitude, particularly when elicited by protocols that rely on strong predictors (Schröger et al., 2015). Such responses can be framed in terms of attentional and contextual enhancement (Schröger et al., 2015) of a stimulus-responsive population whose neural and network dynamic depends on the stimulus history.

This perspective explains why “weak” temporal predictability conditions, like jittered rhythms, can elicit clear omission responses when attention is engaged or multimodal associations are established. Naturalistic or musical stimuli (Janata, 2001; Nemoto, 2012; Bendixen et al., 2014; Lehmann et al., 2016; Vikene et al., 2019) also elicit clear omission responses. Such stimuli might elicit attention, even when unattended, because of their intrinsic relevance. Alternatively, they might have rich, associative representations that recruit the neural mass necessary to generate visible EEG signals (Panzeri et al., 2015).

There are a few cases of animal studies that show omission responses to jittered rhythms (Karamürsel and Bullock, 1994; Prechtl and Bullock, 1994). Omission responses in fish and reptiles were observed with microelectrodes at the early stages of the visual pathway, including at the level of the surgically isolated retina, and closely resembled OFF-responses. These responses are consistent with the rebound from adaptation hypotheses of omission responses, and their detectability does not depend on behavioral modulation as they are an expression of low-level circuitry.

Based on non-rhythmic omission studies, omission responses have been framed as a biomarker of predictive processes (Wacongne et al., 2011). In particular, omission responses are considered a correlate of endogenous, predictive neural activity. Whether such activity is associated with the generation of predictions or rather prediction errors is yet to be determined. The dichotomy between prediction and prediction error might not be clear cut: the empirically informed models of corollary discharge and MMN generation we discussed included forms of prediction and prediction error as overlapping and complementary functions subserved by stimulus responsive microcircuitry. It is thus possible that omission responses are biomarkers of prediction and prediction errors that do not exist on separate units, but rather are the product of a network constantly adjusting its state depending on stimulus history. In fact, Hughes et al. (2001), with intracranial recordings in auditory cortices during an oddball paradigm with omissions, fail to find “veridical” stimulus responses: all recorded responses were driven by stimulation and/or stimulus history, but never by stimulation alone.

Omission responses and MMN are both generated by predictive processes, which in principle could be supported by local auditory circuitry subserving various forms of deviance detection. Such local circuitry could also support corollary discharge mechanisms and sensory suppression, as described by Schneider et al. (2018), with the cortical representation of regularity being controlled by motor projections. If that were the case, protocols that have subjects in control of stimulus delivery, and create an association between an action and the consequent stimulus, should elicit responses to the omission of the associated stimulus when the action is performed.

As a matter of fact, the absence of stimuli that are predictably associated with one’s actions, by paired presentation (Figure 3B), do elicit an omission response (Nittono, 2005; Kühn and Brass, 2010; SanMiguel et al., 2013a, b; Dercksen et al., 2020). These responses to the absence of self-initiated stimuli are strong, easily elicited and detected, and often resemble stimulus-evoked responses. They also present the variety of electrophysiological components at different latencies described for responses to omissions that do not involve stimulus self-initiation. Weaker responses are elicited by unspecific stimulus pairing, i.e., when the features of the regular stimuli are not constant throughout the action-stimulus pairing (Dercksen et al., 2020). These omission responses are obtained with the same protocols that produce action-associated sensory suppression, as described in the context of corollary discharge. Thus, omission responses in self-initiation protocols could be generated by the hypothetical shared substrate we discussed for corollary discharge and MMN. However, at the time of writing, there is no experimental link between human omission responses in protocols with self-initiated sounds and sensory suppression in equivalent protocols.

To our knowledge, only one study addressed the relationship between sensory suppression and omission responses, but only in humans and without the involvement of action (Todorovic et al., 2011). A hypothetical overlap of the substrates of corollary discharges, sensory suppression, omission responses, and MMN can only be tested in animals. Such an investigation would also address the possibility that the omission responses observed in active protocols are not fully cortical: corollary discharges drive sensory suppression also in pre-cortical stations of the sensory pathway, such as the dorsal cochlear nucleus (Singla et al., 2017) and the thalamus (Cavanaugh et al., 2020). These stations have not yet been tested for omission responses. If we hypothesize that sensory suppression for an action-associated sound is complementary to omission responses (when the sound is withheld), then subcortical areas that show suppression could also contribute to omission responses. An involvement of subcortical areas in the generation of omission responses in protocols with self-generated sounds would be consistent with the notion that regularity is processed at multiple levels in sensory pathways, and does not necessarily exclude independent cortical generators (Karamürsel and Bullock, 1999). In fact, cortical sensory suppression appears to be independent of pre-cortical filtering (Todorovic et al., 2011).

A further indication that omission responses rely on mechanisms representing multiple forms of predictability comes from protocols that establish sensory-sensory associations. Studies employing auditory stimulus pairs (Figure 3C; Hughes et al., 2001; Bendixen et al., 2009) detected strong stimulus-like cortical responses to omissions, independent of attention. These responses were elicited only when the second stimulus of a pair (inherently more predictable) was omitted after repeated presentation of the pair. Omission responses are also observed for the absence of stimuli that are predictably associated, by paired presentation, with other stimuli in a different modality (Figure 3D). These responses are elicited when only one of the associated stimuli is presented (McIntosh et al., 1998; den Ouden et al., 2009; Stekelenburg and Vroomen, 2015; van Laarhoven et al., 2017, 2020). Multimodal omission responses consist of activation in the sensory areas in the omitted stimulus modality.

Omission responses in the wide variety of rhythmic and non-rhythmic protocols indicate that many forms of deviation from stimulus regularity are encoded in the auditory cortex, possibly by the same neural circuitry. This hypothesis was presented by Chien et al. (2019) in the framework of a computational model.

## A Computational Model of Circuitry Supporting Deviance Detection

**Figure 4:**
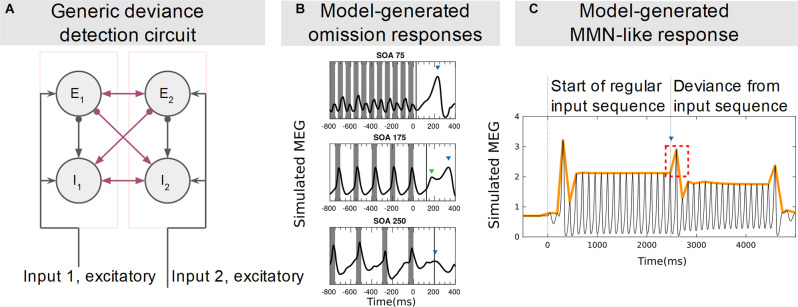
Computational model of deviance detection nodes and simulated MEG signals. **(A)** Architecture of generic deviance detection circuit with two reciprocally connected stimulus-selective nodes (pink squares) that can function as deviance detectors for each other’s activation state. The synaptic weights of the inter-node connections (magenta) are the free parameters of the model. Circles indicate neural populations, excitatory (E_1,2_) and inhibitory (I_1,2_), and lines indicate their connectivity. Arrows and filled circles indicate excitatory and inhibitory synapses, respectively. **(B)** Simulated magnetoencephalography (MEG) signals of a network composed of multiple such nodes include responses (arrows) to the omission (at time 0) of a stimulus from a regular input sequence. The responses, and their dependence on stimulus onset asynchrony, closely resemble those observed experimentally in recordings from human listeners. Adapted from Andreou et al. (2015). **(C)** Simulated MEG signals from a network composed of the same nodes shown in **(A)**. As the input is switched from a regular to a random sequence, an MMN-like increase in response amplitude is observed in response to the first deviant of the sequence (arrow and dashed square). The time series closely resembles those observed in recordings from human listeners (Barascud et al., 2016) with the same stimulation protocol. Adapted from Chien et al. (2019).

The neural mass model (Figure 4) proposed by Chien et al. (2019) accounts for ON/OFF responses, MMN, regarding adaptation and feedforward disinhibition of stimulus-selective populations. The generic deviance detection principle described by Chien et al. (2019) has similar implications to the model of MMN generation proposed by Ross and Hamm (2020) in respect to the processing of prediction and prediction errors. Regularity representation (prediction) is supported by a local network steady-state that primes responses to change in the network’s input (prediction errors) based on local network properties and connectivity. This steady-state may also be modulated by projections from other brain areas, as in the case of corollary discharge. The model itself allows several testable predictions, for instance that NMDA receptor antagonists should affect both MMN and omission responses.

In summary, local circuitry, consisting of multiple neuron types, supporting feedforward and feedback inhibition and excitation of pyramidal output, can detect deviations from regularity (Chien et al., 2019). This pyramidal activity varies with the magnitude and behavioral relevance of the deviation (Schröger et al., 2015). Omission responses might be the result of such deviance detection processes, subserved by interneuron-mediated modulation of pyramidal output. This hypothesis is consistent with the empirical and computational models considered in this review (summarized in Figures 1, 2, 4). They vary in explanatory scope and mechanistic basis, but also share several empirically justified assumptions, which can be condensed in a generalized model, and should be explored with omission protocols.

## Toward A Generalized Model of Cortical Prediction Substrates

All models discussed assume that different stimuli (for instance deviants and standards in an oddball protocol) are represented by different pyramidal populations, depending on their acoustic features. The different populations are interconnected and are also reciprocally connected with local interneurons. The interneurons have inhibitory synapses on similarly and dissimilarly tuned excitatory and inhibitory populations. These reciprocally connected representational nodes respond to stimuli based on their respective activity history, generating responses to change. The node output can be the input for a hierarchy of such nodes, capable of processing more and more abstract forms of regularity and deviation.

Besides connectivity, the production of deviance-specific responses relies on at least two other phenomena. One is short-term plasticity and SSA of excitatory units as suggested by Chien et al. (2019; Figure 4) and Ross and Hamm (2020; Figure 2). The other is the specialized contributions of PV and SST interneurons. PV interneurons provide generalized inhibition under modulatory control, are strongly adapted in conjunction with excitatory units, and are inhibited by SST interneuron activity. SST interneurons in turn are less adaptable and by exerting inhibitory control on PV interneurons, produce feedforward disinhibition of excitatory units. This disinhibition possibly primes NMDA receptor upregulation in non-adapted (deviant-responsive) populations, thus promoting deviance and omission responses. PV inhibition by SST interneurons might also be responsible for the reduction of unspecific sensory suppression during movement after a sensorimotor association is acquired. In fact, modulatory input from cortical projection neurons in other modalities is also received by representational nodes, possibly affecting the same inhibitory and excitatory populations involved in the generation of MMN. The modulatory input could be either interneuron-specific or also target excitatory units. Hebbian plasticity at the target sensory interneuron synapses guarantees the synaptic association of the modulatory input and the representational units, affecting the local representation of regularity.

An important notion to test is whether these nodes undergo constant endogenous activation and modulation, which would be revealed by omission responses. The heterogeneity of results from omission studies employing methods with different sensitivity may stem from the modulation of these nodes by motion, multimodal associations, attention, behavioral state, and other ongoing sensory processes. Alternatively, the heterogeneity of omission responses could indicate unrelated, non-overlapping mechanisms. A systematic model-driven investigation of omission responses is necessary to differentiate between these options. Opto- and chemogenetic manipulation of selected neuronal populations at different levels in the sensory pathways, in combination with stimulus omission, will allow the assessment of whether stimulus-specific populations act as deviance detectors. If the proposed models are accurate, the comparison of omission and mismatch responses should reveal two kinds of activity: focal activity of a stimulus-selective population at deviant onset (mismatch only) and distributed activity of multiple such populations at the time the standard stimulus is due (mismatch and omission). Furthermore, these techniques can be employed to assess the contribution of different processing stages along the sensory pathway to deviance detection phenomena in the cortex. Another important experimental step is the manipulation of NMDA receptors with proper controls to assess their role in predictive processes. In particular, whether they are implicated in both omission responses and MMN, specifically for regularity formation rather than deviance detection as predicted by Chien et al. (2019), and whether they are prominently involved in deviance detection rather than SSA, as discussed in Ross and Hamm (2020). Such evidence could reinforce the hypothesis that both phenomena are subserved by shared mechanisms.

## Conclusion

In conclusion, model-mediated interaction between human and animal research is informative concerning the cortical generators of MMN and corollary discharge. The underlying circuitry does in principle support omission responses as well. Biologically inspired computational models allow the translation from circuit activity measured in animals to the macro-level responses observed in human studies. Animal implementation of cognitive neuroscience protocols, in conjunction with cell-type-specific imaging methods, is thus necessary to determine the substrates of deviance detection phenomena. These methods have already demonstrated the importance of feedforward inhibition in the generation of mismatch responses. It is necessary to assess whether this and other deviance detection phenomena, like corollary discharge and omission responses, are generated by the same local cortical circuitry, or by similar but non-overlapping networks, or by unrelated mechanisms. Re-use of the same circuitry is consistent with the current understanding of cortical structure and function, and its conservation across species and modalities. Among deviance detection protocols, omission protocols allow a focus on local circuit activity and its top-down modulation by evoking deviance detection in the absence of sensory input. The silencing of bottom-up input is an important tool to dissect neuronal circuits and to explore the substrates and empirical consistency of prediction and prediction error, the main actors of predictive processing theories. Interactionist investigation of cortical circuitry across species with omission protocols would provide the biological foundations of core concepts in the predictive processing framework and an empirical test of the framework’s unifying potential. These are important objectives, given that predictive theories of brain disorders are increasingly influential in the clinical community. A substrate-level understanding of their mechanisms is necessary to make these theories empirically testable and to open new avenues of diagnosis and treatment.

## Author Contributions

All authors contributed equally to the article and approved the submitted version.

## Funding

The authors acknowledge support from the German Research Foundation (DFG) and the Open Access Publishing program of the Leipzig University.

## Conflict of Interest

The authors declare that the research was conducted in the absence of any commercial or financial relationships that could be construed as a potential conflict of interest.

## Publisher’s Note

All claims expressed in this article are solely those of the authors and do not necessarily represent those of their affiliated organizations, or those of the publisher, the editors and the reviewers. Any product that may be evaluated in this article, or claim that may be made by its manufacturer, is not guaranteed or endorsed by the publisher.
